# Aesthetic perception and its minimal content: a naturalistic perspective

**DOI:** 10.3389/fpsyg.2014.01038

**Published:** 2014-09-19

**Authors:** Ioannis Xenakis, Argyris Arnellos

**Affiliations:** ^1^Department of Product and Systems Design Engineering, University of the AegeanSyros, Greece; ^2^The KLI Institute for the Advanced Study of Natural Complex SystemsKlosterneuburg, Austria

**Keywords:** aesthetic perception, emotions, anticipation, perceptual content, interactive affordance, agency, normativity

## Abstract

Aesthetic perception is one of the most interesting topics for philosophers and scientists who investigate how it influences our interactions with objects and states of affairs. Over the last few years, several studies have attempted to determine “how aesthetics is represented in an object,” and how a specific feature of an object could evoke the respective feelings during perception. Despite the vast number of approaches and models, we believe that these explanations do not resolve the problem concerning the conditions under which aesthetic perception occurs, and what constitutes the content of these perceptions. Adopting a naturalistic perspective, we here view aesthetic perception as a normative process that enables agents to enhance their interactions with physical and socio-cultural environments. Considering perception as an anticipatory and preparatory process of detection and evaluation of indications of potential interactions (what we call “interactive affordances”), we argue that the minimal content of aesthetic perception is an emotionally valued indication of interaction potentiality. Aesthetic perception allows an agent to normatively anticipate interaction potentialities, thus increasing sense making and reducing the uncertainty of interaction. This conception of aesthetic perception is compatible with contemporary evidence from neuroscience, experimental aesthetics, and interaction design. The proposed model overcomes several problems of transcendental, art-centered, and objective aesthetics as it offers an alternative to the idea of aesthetic objects that carry inherent values by explaining “the aesthetic” as emergent in perception within a context of uncertain interaction.

## INTRODUCTION AND MOTIVATION

Before explicit aesthetic judgments emerged in human culture, individuals did not necessarily discriminate objects^1^ as exhibiting a type of *rightness–* a “proper” organization of features^2^ *–*in their structure from other objects that did not (Beardsley, 1975). However, from a phenomenological point of view, there was at least an unspecified *interest* in some objects rather than others. The problem of rightness was introduced when scholars in the humanities attempted to explain our interest in some objects (rather than others) in terms of aesthetic values and properties that are connected to “ideals” about beauty and ugliness, which provoke pleasure and displeasure, respectively, in a perceiver. These special objects are connected to a superior reality, one that demands exceptional cognitive skills in order to be properly grasped. This is a tradition that persists from Plato to the contemporary literature on aesthetics, and was re-enforced when Kant intoduced the term of “disinterestedness” so as to express the state under which this exceptional form of perception is possible. For Kant (1998, 2000) and his followers, the experience of the transcendental aesthetic is possible only when, during perception, someone can remove any conceptual (e.g., purposive intention, an interest that may serve an instrumental or ulterior purpose) and sensual processes, so that one has only a pure intuition and the mere form of appearance. When the above conditions are not met, the object cannot be perceived aesthetically, which suggests that it was not an aesthetic object.

Current practitioners of the above “art-centered” approach still maintain a sharp distinction between works of art and everyday objects that afford aesthetic and common perceptions, respectively. In the last few years, models and explanations from other scientific fields, which traditionally investigate cognitive behavior, have been added to this approach. While fields such as interaction design and psychology offer promising new methods and tools to study “the aesthetic” beyond transcendental explanations, in many of these writings we can still see a strong influence of the art-centered approach (see, e.g., Berlyne, 1971). This is quite evident in several contemporary studies that by following a basically externalist account of aesthetic perception aim to determine what organizations of features elicit aesthetic responses (see, e.g., Locher, 2003; Bar and Neta, 2006; Noble and Kumar, 2010). Even several works in neuroscience in which authors attempt to ground and explain aesthetic perception in terms of specific bio-cognitive functions subscribe to an externalistic perspective as well, as the neuroscientific experiments envisaged focus almost exclusively on art-centered interactions (e.g., see Kawabata and Zeki, 2004; de Tommaso et al., 2008; Mallon et al., 2014).

The perspective we will defend is quite radical in that it departs from traditional but also from several contemporary philosophical conceptualizations of “the aesthetic” by opposing the triad of transcendental, objective and art-centered aesthetics. The “aesthetic” etymologically originates from the Greek word αισ𝜃ητικóς (aisthētikos), meaning “the one who pertains/deals with the senses,” which was derived from the word αισ𝜃 ανoμαι (aisthanomai) meaning “I perceive, feel, sense.” On the same grounds, Baumgarten introduced the term “aesthetics” as denoting the fundamental cognitive task of knowing things through the senses (Beiser, 2011). Nevertheless, according to Beardsley (1969; 4), who is considered as one of the most important late 20th-century aestheticians, “Whatever its origin, this concept undoubtedly achieved its fullest development and its richest application in the aesthetic theory of John Dewey.” According to Dewey (1980, p. 16) the experience of “the aesthetic” involves a “drama in which action, feeling, and meaning are one.” For Dewey (1980, p. 19) “experience is the fulfillment of an organism in its struggles and achievements in a world of things, it is art in germ. Even in its rudimentary forms, it contains the promise of that delightful perception which is aesthetic experience.”

Dewey, then, argues that art and aesthetic experience are not limited to the usual artistic objects but extend to all expressions of life. Accordingly, our main aim in this paper is to investigate the possible conditions under which aesthetic perception and its minimal content is produced in all those expressions of life, and particularly in everyday interactions beyond art that are not commonly regarded as aesthetic. If one accepts that there are no aesthetic objects *per se*, then an object is considered aesthetic with respect to the interaction context in which it is perceived. Also, since “the aesthetic” cannot be found in the object, those objects that are nowadays widely considered aesthetic *per se* did not pre-exist but the interactions with them have developed to the point that we now consider these objects as such. In other words, *there is no such thing as an aesthetic object per se, but it is a particular interaction that might have an aesthetic dimension or not.* As Dewey (1980, p. 12) said, “the answers cannot be found, unless we are willing to find the germs and roots in matters of experience that we do not currently regard as aesthetic.” Hence, we are interested in those “aesthetic” interactions and particularly in the type that fosters the emergence of *feelings* that promote the construction of a potential preference in everyday objects or state of affairs, and which cannot be dealt with in the triptych of transcendental, objective, and art-centered aesthetics. In this sense, there are several everyday cases of interaction that most aestheticians do not regard as “aesthetic” but in our view involve *feelings of a potential preference* on the basis of which the “germs and roots” of aesthetics are developed. These cases clearly illustrate that “the aesthetic” is not a feature of the object. For instance, some people feel they can be very productive when they find themselves in chaotic workplaces (e.g., Francis Bacon in his art studio), while others feel they are completely unable to accomplish any task in such interactive (working) contexts. A car that the designer expects to cause a tremendous thrill is perceived differently in Western civilization and by indigenous people. In each of these cases *feelings* may influence a potential preference that may in turn become a judgment; or, for whatever reason, these feelings can be modified during interaction. For example, users may expect using a high-tech coffee machine to be the best way to prepare a coffee, but they can change their mind during the procedure. Someone could start feeling anxious and uncomfortable during a film after having decided to watch it on the basis of positive critiques from prior viewers who enjoyed it. Moreover, a situation that is undoubtedly “aesthetic” for an art-centered aesthetician may not be perceived as such. For instance, some people do not, cannot, or refuse to interact aesthetically with a famous painting, while others can be totally indifferent independently of the musician performing a well-known piece of music (see, e.g., the *Washington Post’s* experiment with Joshua Bell). Moreover, the same agent can interact with the same “object” (or state of affairs) through different *feelings*. For example, someone who originally expected to spend a wonderful time as a tourist on a island may later start feeling that it was a bad idea to spend the rest of her life there. In all these cases of interaction the germ of “the aesthetic” is developed when subjective expectations are influenced by these *feelings* produced during the agent’s attempt to assign meaning and understand the implications that a situation would have for its goals. Undoubtedly, the list of such everyday interactive cases is endless, since a changing situation is a new opportunity for the agent to cope with by developing feelings of various intensities that influence the construction of the agent’s meanings and consequently the management of interactions.

Our aim is not to explain all these phenomena completely. This would be an extremely ambitious task given that aesthetic perception is a multidimensional phenomenon involving several different aspects of cognition and action (see, e.g., Leder et al., 2004). Accordingly, we do not aim to explain what an agent specifically likes or dislikes, and we don’t deal with the outcome of particular aesthetic judgments or judgments of preference. We rather suggest that all phenomena pertaining to “the aesthetic” should be dealt with in a different theoretical and explanatory context, the theoretical and conceptual basis of which we attempt to provide here.

For naturalists such as Dewey and James, aesthetic perception, as any other type of perception, presupposes interaction. Dewey (1929) has very effectively argued that perception is an acknowledgment of unattained potentialities for interaction^3^. To perceive is to refer the current situation to consequences and to act accordingly. This means that perceivers are neither spectators nor passive recipients of information from the environment. Rather, they are cognitive agents that interact intentionally in their environments. Thus, naturalists deny the possibility of disinterested interactions. Perception is thus a predictive expectancy. For Dewey, the potential consequences of interaction are the sense that an object makes. This sense is a product of intellect and inference usually associated with an aesthetic intuition, where feeling, understanding, and action are one. As Dewey (1980; 15) has stated “In a world like ours, every living creature that attains sensibility welcomes order with a response of harmonious feeling whenever it finds a congruous order about it.” In other words, when the consequences of potential interaction are evaluated as promising (anticipating) order or disorder, their perception is aesthetic in nature. In all, aesthetic perception contributes to sense making^4^.

These aesthetic evaluative outcomes are evoked through the emotions associated with the bodily and behavioral changes that occur during an interaction (Shusterman, 2013). Indeed, all relevant theoretical explanations and models, and all relevant experimental studies suggest that all activities that are eventually deemed *aesthetic* involve emotional processes of the same type and mode of realization as those that influence and prepare an agent to act (see, e.g., Chatterjee, 2011). The functional overlapping of processes related to aesthetic responses to works of art with those pertaining to emotional evaluation in any adaptive perception suggests that “the aesthetic” does not pre-exist (at least not in the object itself), but on the contrary it emerges in perception during interaction.

To sum up, in a naturalistic perspective “the aesthetic” should be conceptualized and investigated as related to meaningful patterns of activity, and therefore to action selection, and consequently to the construction of preferences regarding the interaction with certain objects, which in turn could be objects that we end up *liking or disliking*^5^. Therefore, in this paper we are interested in providing the conceptual and theoretical basis to explain the emergence of *feelings* that promote the construction of a potential preference or interest in everyday objects or state of affairs, and which cannot be explained in the externalist and art-centered context of rightness. In accord with Rolls (2011), we claim that such emotional feelings could emerge in every interaction under the appropriate conditions, and they could be the origin of several types of aesthetic judgments. It should be noted that these feelings can only partially influence the agent’s actions or judgments in the given conditions. The proposed model does not cover all other factors and aspects (e.g., rational inferences, learning, acting by habit or by analogy making, etc.) that influence an agent’s judgments, and which provide other values influencing sense making and the respective content. We deal with the minimal content of aesthetic perception. Accordingly, we here deal neither with beauty and ugliness nor with the modeling of such judgments. More specifically, we do not deal with whether designed objects like a car, a mailbox, a coffee machine, a dinner table, the cockpit of a jet airliner, a work of art or objects of nature (e.g., a landscape, or other human beings and animals) are beautiful or not. Actually, according to the perspective offered here we completely dismiss such a question. What we are interested in is to provide the theoretical basis to understand the general characteristics and requirements of the interaction context within which agents aesthetically perceive or feel like that is possible (or tend) to engage with the above things in specific interaction contexts by following these feelings. We will argue that such emotional feelings add an aesthetic dimension to every interactive situation, and consequently to the respective objects.

The structure of the paper is as follows. In section “Aesthetic Perception and Objects” we show that an understanding of aesthetic perception as intrinsically embodied in the “aesthetic object,” or as belonging to unintentional forms of perception, cannot provide a model of aesthetic perception that will satisfactorily reply to the issues raised above. In section “Aesthetic Perception in the Context of Interactivism and Embodied Cognition” we develop a model of aesthetic perception that is consistent with living agency, particularly with its *normative* characteristics. In section “Perception Within a Normative Account of Sense-Making” we focus on the necessary organizational requirements for agency at a level that supports perception through the construction of emotionally based meaningful patterns of activity. Then, in section “Aesthetic Emotions and Their Role in Sense-Making” we provide neurophysiological evidence of human agency in order to support our claims for the respective organizational aspects. Finally, in section “A Model for Aesthetic Perception and Its Minimal Content” we suggest that aesthetic perception, through its minimal content, has an adaptive preparatory and anticipatory nature, the normative function of which is to evaluate, through emotions, the appropriateness of a potential interaction^6^. Our view is mostly based on contemporary theories and explanations in the realm of interactivism and embodied cognition, contemporary evidence from emotional theory, neuroscience, and findings from relevant studies in the experimental aesthetics and interaction design.

## AESTHETIC PERCEPTION AND OBJECTS

### THE AESTHETIC OBJECT AND ITS RIGHTNESS

This section discusses the common view that aesthetic perception bears an objective part in its content, one that enables the categorization of some objects as aesthetic. Quite often in the literature, aesthetic objects are considered all those things that “*carry a certain function”*: *to provoke an aesthetic form of response, to transmit information that evokes an aesthetic interest or experience* (see, e.g., Ingarden, 1961; Walsh, 1974; Lind, 1980; Carroll, 1986; Bennett, 2002). However, there is an unclear assumption in this definition. Even though it is accepted that the object is subjectively evaluated in experience, authors do still believe there is somehow a kind of “objectivity” in perception. From this perspective, the object carries something *special* within its properties, some *perceptual features*, the communication of which could explicitly relate the object to aesthetic forms of response (see, e.g., Leder et al., 2004; Tilghman, 2004; Brandt, 2006; McManus et al., 2011). In this respect, aesthetic perception depends on the detection of “rightness,” the formalization of which is associated with an *aesthetic property.* Rightness and aesthetic properties may have positive or negative values. Their perception is determined by a “proper” organization of non-aesthetic features but cannot be reduced to them (see also Levinson, 2005, 2011). For instance, something is “garish” when it has a pattern of bright colors, but bright colors are not aesthetic *per se*; they are just pigments. They constitute “garish” only when they are applied “properly.” However, “garish” has usually a negative meaning, especially for those for whom bright colors are not their style. Notwithstanding this, the application of the “aesthetic in objects,” especially since the Bauhaus school, was nevertheless related to fixed norms or hardwired principles that guide designers and artists in formalizing their sense of rightness into aesthetic properties (Kim, 2006). This was for years a “safe” strategy to place the object in the category of “the aesthetic.” Following this mostly institutional strategy several scientific fields have attempted to objectify aesthetics. Taking into account the impact specific organizations of features may have in aesthetic perception (see, e.g., Silvera et al., 2002; Bar and Neta, 2006), several studies aim at measuring them, and objectifying their design by proposing them as aesthetic indexes (see, e.g., Jacobsen et al., 2006; de Tommaso et al., 2008). However, studies that test the validity of these general laws have not yielded satisfactory results with respect to the establishment of a general explanatory model of aesthetics (Leder et al., 2004). In general, empirical aesthetics, based on an art-centered tradition, has difficulty modeling and generalizing aesthetic guidelines even for simple interactive cases dealing with relatively simple forms of organization of features^7^. In addition, experimental findings indicate that what is considered as right is subjective and not always perceptible by everyone, since perceptual content is dynamically influenced by personal meanings and values (Locher, 2003) as well as by the context of interaction (Blijlevens et al., 2012). Moreover, experimental evidence suggests that aesthetic perception is mostly related to one’s *idea of rightness* as an optimal organization that cannot be reduced to a specific placement of features within that organization (Locher et al., 1999). The subjectivity in the communication of rightness is compatible with contemporary approaches to the “design process^8^.” The communication of rightness concerns both those who design an experience through a medium (design, artwork, etc.), and those who are engaged in an experience through this medium (Arnellos et al., 2010a). Designers seek to formalize their own *idea of rightness* into a designed medium by choosing out of unlimited possibilities those that are anticipated to engage perceivers and provide an optimal interaction (Xenakis and Arnellos, 2012). Perceivers should similarly detect the designer’s *idea of rightness* in these mediums or to perceive *their own rightness.* The latter may correspond to personal optimal ways of interaction that hardly coincide with a designer’s ideas. Moreover, an understanding of aesthetics, focusing on the perception of specific properties that constitute rightness does not explain the content of aesthetic perception. Especially when perceivers cannot see the designer’s *idea of rightness*, they see *their own rightness* or they do not care about rightness (Frohlich, 2004). Hence, if we accept the idea that something “transforms” an organization of non-aesthetic features to aesthetic properties it seems that this transformation is not due to the object itself but rather due to perceptual processes realized in a context whose characteristics and properties are still in question.

Kantian *disinterestedness* (Kant, 2000) provides a possible explanation to this “special” context within which an agent perceives something aesthetically or not. According to Kant, emotions of pleasure and pain involved in aesthetic perception are unintentional (free from any kind of desire, aim, or purpose, or any social, moral, or intellectual considerations), in contrast to intentional reasonable thoughts involved in non-aesthetic perception. Influenced by the above difference, several authors approach “the aesthetic” as a property that has a vague relationship, or no relationship at all, to more *pragmatic* features that are associated with an object’s perceived usability, functionality, and instrumentality^9^ (see, e.g., Brandt, 2006; Hassenzahl, 2008; Moshagen and Thielsch, 2010). While objectives of utility and functionality are always designed and perceived intentionally (Crilly et al., 2009), the design and perception of “the aesthetic” lack such characteristics. Traditionally, in the design process “aesthetic phenomena seem apparently useless” as their perception emerges unintentionally during interaction (Hekkert, 2006, p. 161; see also Hekkert and Leder, 2007). This argument is quite radical, if not simply untenable, when seen in a naturalized context. Nevertheless, it still does not devoid “the aesthetic” from its normative dimension. Normativity plays a fundamental role in sense making and more generally in the characterization of agency in living systems (Barandiaran and Moreno, 2008; Burge, 2009; Arnellos et al., 2010b; see Christensen, 2012 for further analysis). As we argue in section “Aesthetic Perception in the Context of Interactivism and Embodied Cognition,” agents use their emotions normatively and produce meaning from their environment, even when these agents apparently remain inactive or seem to have no relation with their physical and social environments (Damasio, 1995). Moreover, it is now accepted among emotional theorists that emotions are not elicited in isolation and that they play an important role in sense making, even though agents may not be fully aware of those processes (see, e.g., Baumeister et al., 2007; Rolls, 2011). In addition, several studies have shown an unexpected correlation between aesthetics and instrumentality during the overall experience with objects (see, e.g., Tractinsky et al., 2000).

Returning to our initial inquiry concerning the possibility that aesthetic perception bears an objective part in its content, one which is intrinsically related to features, we could conclude that it is naïve to think that “the aesthetic” is pre-given to perception as a ready-made element reduced to specific organizations of features (Mitias, 1982). On the contrary, as we discuss in the next section, other aestheticians who aim at specifying the context in which one perceives an organization of non-aesthetic features as aesthetic properties argue in favor of an anticipatory content of an object.

### THE ANTICIPATORY CONTENT OF AESTHETIC PERCEPTION

We focus on two theoretical problems relevant to our purposes. The first one concerns the facultative nature of all those mechanisms producing an aesthetic perception, while the second concerns the understanding of the relation between those mechanisms and the “proper” organizations of features usually characterized as aesthetic properties. The second problem is related to the hypothesis that the perception of aesthetic properties is a category distinct from any other ordinary form of perception.

According to contemporary aestheticians (e.g., Sibley, 1959, 1965, 2001; Carroll, 1999; Matravers and Levinson, 2005; Levinson, 2006, 2011), aesthetic properties are mostly proposed to be *higher-order ways of appearing*. Examples may include (but are not limited to) properties such as delicate, graceful, unified, integrated, and so on. Sibley and Levinson argue that the perception and understanding of these higher-order ways of appearing is emergent and cannot be reduced to specific organizations of non-aesthetic features, known as *lower-order ways of appearing* (e.g., colors, textures, timbres, etc.). More specifically, a higher-order way of appearing emerges from lower-order ways of appearing as a result of emotional and conceptual processes. Thus, these lower-order ways are always open to interpretation. While this argument seems valid from the point of view of semantics and concept formation, it does not explain which part of the interactive process alters an ordinary interpretation into an aesthetic one. If there is a distinction between aesthetic and non-aesthetic properties, then there must be an as yet unspecified aspect during perception that guides perceivers exclusively to aesthetic forms of response.

Matravers and Levinson (2005) try to overcome this predicament by suggesting that aesthetic properties pull their metaphysical weight into some *explanatory value* in objects, “in the sense both of explaining the experience we have of them and of explaining the normativity that attaches to attributions of them” (p. 214). What they propose is that beholders are motivated to assign *explanatory values* to objects so as to specify those things an *object could offer* them during an experience. In other words, aesthetic properties become *ways of being that are closely related to possibilities of a future state*, and such possibilities are evoked from the appearance of the object.

However, this does not explain the facultative nature of the evaluative process, which motivates perceivers to use values during aesthetic perception. As we discuss in section “Aesthetic Perception in the Context of Interactivism and Embodied Cognition,” the framework for concept formation used by Matravers and Levinson applies to all the ways in which agents are engaged in meaningful interactions with their environments. In a naturalized context of interaction, agents make sense of their environment by assigning values to possibilities of future states according to their motives and goals (Christensen, 2012). So, what distinguishes a value that serves an agent’s adaptive perception from an aesthetic one is still in question, while the allegedly special aspect, which guides perceivers exclusively to aesthetic forms of response, remains unspecified. Quite recently, Sauchelli (2013) has claimed that adaptive goals may influence a perceiver’s aesthetic perception. In particular, Sauchelli proposes that “proper function,” which he considers a function that is known for accomplishing the goals an object is designed for, may influence a perceiver’s expectations and, consequently, the aesthetic perception itself. These expectations for Sauchelli are beliefs “concerning how a certain function should or will be realized in a form in order for the resulting object to be suited to its function” where “beliefs are the result of our previous experiences with objects appearing to have the same (or similar) functions” (p. 46). Sauchelli considers a certain range of aesthetic judgments – those that involve only functional aspects. However, as he argues, his theory does not imply that beauty (whatever the term might means) should be explained solely in terms of function (as utility) or that ascriptions of beauty should always be grounded in expectations. Sauchelli, then, allows for the consideration of other forms of “aesthetic perception” that are beyond this category of functional interactions. Moreover, his aesthetic theory is based on anticipation, which presupposes established knowledge and experiences. As we argue below, the emergence of the aesthetic perceptual content may benefit from previous experiences but does not presuppose their existence. For instance, agents do not need to know how football shoes function in order to anticipate an optimal “football play.” Most of the time users are incapable of detecting scientific improvements in a football shoe that will provide them with an advanced control of the ball. Their evaluations are thus based on a great number of goals/standards/criteria that constitute their personal norms. For example, none of this scientific information is useful (or relevant) when the agent’s goal is to provoke other players or spectators to turn their eyes to his feet during a play (including cases where the agent is not a good player). Thus, different goals provide to the agent different evaluative frameworks under which the shoe is perceived and probably used. This is why football shoes designers do not limit their ideas to shoes that provide only a good control of the ball, but rather attempt to design the whole experience including all those social implications that affect our interaction with products.

Quite interestingly, Beardsley (1970) under Dewey’s influence uses the terms “commodity” and “firmness” in a manner reminiscent of Sauchelli’s “proper function.” *Commodity* is about function and refers to features that make objects appropriate (proper) for what they are designed for, while *firmness* is about construction and refers to the *right* organization of features in an object. Specifically, Beardsley argues that good engineering, good organization, and usefulness are factors that improve people’s lives and lead beholders to perceive an object from “an aesthetic point of view.” Accordingly, perceivers assign aesthetic values to objects because they are in position to detect a specific value in them according to their interests. When somebody estimates an aesthetic value, she forms an expectation or anticipation that the estimated result will conform to or promote her personal (aesthetic or not) goals/standards/criteria (see pp. 44–45).

Therefore, anticipation in aesthetic perception implies an evaluation mechanism influenced by criteria that help perceivers to evaluate an object on the basis of those aspects that will somehow be fulfilled when the object is used in the future. Because of this, the content of positive aesthetic evaluation is related to an anticipation of pleasure that the object could *offer* to the perceiver in accordance with her goals/standards/criteria. Thus, “the aesthetic” is evoked in perception, when an *offer of goal fulfillment* is obtainable in the object in the sense that its organization could support an interpretation of what the perceived object might *afford or offer*.

Our aim is to elaborate the above conceptualization of “the aesthetic” into a normative account of perception by proposing a model that aims at describing and explaining the facultative nature and the interactive properties of the underlying embodied mechanisms. In the next sections we discuss some general but key characteristics of a naturalistic framework of perception based on contemporary theories and explanations in the realm of embodied cognition and interaction, with a focus on the role of emotions in the anticipatory preparation for sense making. The proposed model of aesthetic perception is also compatible with contemporary evidence from emotional theory, neuroscience, experimental aesthetics, and interaction design.

## AESTHETIC PERCEPTION IN THE CONTEXT OF INTERACTIVISM AND EMBODIED COGNITION

The naturalistic context in which we attempt to explain the aesthetic perception opposes any dissociation between perception and cognition, according to which cognition diverges from perception as a non-perceptual faculty or conversely, perception diverged from cognition focusing on bottom-up sensory mechanisms and ignoring top-down effects. Our arguments are based on evidence suggesting that perception and cognition share common bio-cognitive systems, which are simultaneously operating in the same mechanisms (Barsalou, 1999). This does not mean that perception and cognition are identical, but rather they share content during sense making.

### PERCEPTION WITHIN A NORMATIVE ACCOUNT OF SENSE-MAKING

Agency presupposes normative interaction. It concerns the ability of the agent to interact with its environment by taking the initiative based only on its self-defined norms (Arnellos et al., 2010b). In this way, agents should always at least try to select which of the interaction alternatives are the best to engage in (Seth, 2007). This initial urge for selection is what generates meaningful patterns of activity. *Selecting the best alternative* does not refer only to those interaction alternatives that preserve an agent’s viability, but also to those ways of interaction that will increase its wellbeing. As we shall see, such selection implies an agential organization that enables the agent to direct itself toward those *indications*^10^ of interaction that will better satisfy its norms.

For such selection to be possible, all alternatives (in some functional sense) should be indicated as available potential interactions. The relation between an indication (e.g., a traffic jam in the highway) and its interaction potentiality (e.g., the agent alters its course) refers to the *consequences* of this indication for the agent (see Bickhard, 1993) and presupposes an agential organization that supports *evaluation* mechanisms that are able to characterize the indicated situation as potential or not. Thus, interactive indications have an anticipatory nature, and if selected, they can indicate true or false, right or wrong (Bickhard, 2006). Based on these norms, the agent can detect the error in case the action does not yield the anticipated outcome. Now, it should be noted that indications *are sufficient to the potentiality of what they indicate* (Bickhard, 1993; 301). In other words, there could be several other indications of the same interaction potentiality, and/or the agent could be in other interactive conditions than the one anticipated. Thus, an indication *per se* does not specify a feature of the environment, but rather provides the agent a normative way (through its organization) to determine if such indication holds or not, while all these indications have always the possibility of failure (Bickhard, 2000). This possibility of failure is what introduces *uncertainty* in interaction (interaction uncertainty), in the sense that the agent is *uncertain about the consequences* that the interactive indications may have with respect to its norms (see also Xenakis and Arnellos, 2012).

A normative account of meaning-based interaction presupposes, then, that agents set their own norms (either implicitly or explicitly) and form meaningful patterns of activity accordingly. They do this by directing and *evaluating* indications with respect to these norms. According to their capacity, agents can construct new norms, which can be modified in the future. These could be general norms stemming from generalized preferences that provide normative orientation with respect to widely integrative aspects of wellbeing (Christensen and Hooker, 2000). A generalized norm could indicate *the optimal way of interaction*^11^. This could be used as a reference point by which agents evaluate their interactions.

To sum up the arguments made so far, for an agent to be able to direct itself toward those interactions that will better satisfy its norms, the agent should exhibit the capacity to differentiate between situations in the environment. Situations (or relations) are differentiated by their interaction potentialities in the sense that in each situation the agent anticipates the realization of those actions that will better satisfy its norms. Thus, a normative account of sense making is future-oriented and has a *preparatory* and *anticipatory* character, which is also subject to dysfunction or failure (Skewes and Hooker, 2009; Bickhard, 2011). The respective interactive-feedback loop provides the agent with the capacity to learn from its failure, and consequently, the possibility for a more elaborated adaptive interaction in the future is increased (Christensen and Hooker, 2000; Bickhard, 2006; Arnellos et al., 2010b). Adaptive agents thus actively construct a *relation* between them and their world. Certainly, this relation is not pre-given but emergent and evoked during interaction. It thus constitutes a sense-making process in which the agent actively constructs a body-mind-environment relation (Thompson and Stapleton, 2008).

In the context of sense-making sketched above, perception is considered a process by which agents are enabled to functionally detect all those interactive indications that could form their *relation* with the world. Therefore, perception refers to a web of ways of interaction, which constitutes the current *situation*. In such a naturalistic but non-reductionist account, perception has the normative function of signaling the agent for both internal and external modifications that will allow the agent to further its purposes (Merleau-Ponty, 2002). Particularly, *perception* functions as a preparatory-anticipatory process, through which agents monitor and manage the respective interoceptive and exteroceptive inputs by *evaluating* them with respect to the consequences they have for their norms. Therefore, *the content of perception is normative, and should be understood with respect to the interaction.* More specifically, *perceptual content is the emergent outcome of an evaluation process, through which the agent is facilitated in constructing its relation with the world by selecting the best indications of interaction according to its norms.* Objects are thus perceived as *evaluated indications of potential interactions*, otherwise agents could not make sense of these objects (Nanay, 2012; Xenakis and Arnellos, 2013). Since the agent always updates its situational knowledge, the situation is not static and the content of perception is itself dynamic (Bickhard, 2009). Such updates may be based on an agent’s inner state, the whole or parts of the situation, properties of objects in the situation, objects in certain relations in the situation, or all the above (Burge, 2005). In accordance with the preparatory-anticipatory character of perception, its content would not always imply a successful interaction outcome, but its occurrence seems necessary for any contribution to the world.

Within this naturalistic account of perception, aesthetic perception could not be a distinct kind, or a perception plus something aesthetic. As any other form of perception, *aesthetic perception should have a normative content* through which the agent actively relates itself to the world. In addition, aiming at a naturalized explanation concerning aesthetic perception, we need to consider such perception as a process grounded in the agent’s organization, just as any other bio-cognitive process that serves the agent’s adaptability and wellbeing. As we suggest in the following sections, a naturalistic account of aesthetic perception should be grounded on emotions.

What we have argued so far is that *the content of perception* emerges when the agent detects and evaluates *indications of potential interactions,* namely, the actions being available to the agent at the exact time of interaction (Bickhard, 2011). As mentioned before, by directing and evaluating these indications the agent is able to *prepare* itself, to anticipate the outcome of the interaction, and to act accordingly. Therefore, during perception every object is evaluated with respect to the range of potential interactions it *could afford in the current situation*.

This notion of “*evaluated indications of potential interactions*” is reminiscent but not fully compatible with the Gibsonian conception of “affordances.” More specifically, in accordance with Gibson (1986) we also conceive affordances as possibilities for action that denote a relation between properties of the environment and of the agent. However, contrary to our view, for Gibson (1986; 127) affordances are based on the idea that “values” and “meanings” of things can be directly perceived in the sense that such values and meanings are external to the perceiver. This makes affordances both objective, real, and physical (unlike the orthodox conception of values and meanings) and at the same time subjective since they refer to the agent. The values and meanings of affordances are specified in the structure of ambient light as information with which agents establish perceptual content. For Gibson affordances are invariant combinations of ecological properties of things taken with reference to the agent’s biological and social needs as well as its action-systems and its anatomy. Although Gibsonian affordances refer to agents, they are permanent in the sense that they do not change as the needs of the agent change. This means that even though an agent’s preferences could fluctuate, (something that the agent likes today may look bad tomorrow) affordances stay invariant. Affordances do not cause behavior but constrain or control it (Gibson, 1982; 410–411). Now, apart from these apparent differences to our framework, Gibson (1986; 143) talks about basic affordances of the environment, which are usually perceived directly, without an excessive amount of learning. However, as Shaw (2003; 82) has claimed there are interactive cases “where Gibson allowed perception to be indirect where paintings, photographs, TV, movies, virtual realities, and other facsimiles of reality are concerned, although he strongly opposed the traditional attempts to reduce all perception and cognition to being indirect, that is, mediated by memorial states or inferential processes.” This is probably a reason why Gibson says that aesthetic objects are different cases and the respective “problems of aesthetics exist in their own right” (Gibson, 1986; 291). As Gibson (1986; 273) has stated:*“We can distinguish between a surface as an aesthetic object and a surface as a display of information. The surface that displays information may also be an aesthetic object, but the cases are different. A picture is a surface that always specifies something other than what it is.”*

Our view of affordances is more akin to Chemero’s (2003); affordances cannot be properties, or even features, of the environment alone. Affordances are features of whole situations and this whole supports (perhaps demands) a certain kind of action. Affordances for Chemero are not in the environment but rather they are relations between the *abilities* of an agent and features of the environment: “An animal typically perceives only the affordance relation, though, and not the constituent relata.” (p. 191) Chemero clearly aims at a normative explanation of affordances. The normative aspect in his explanation for affordances as *relations* is based on the agent’s abilities. As he states, agents with abilities are supposed to behave in particular ways, and they may fail to do so. This explanation puts the normative aspect of the perceptual content in the agent and not externally to it.

However, based on our claims in the beginning of this section, our model of the perceptual process differs in some respect from Chemero’s explanation. More specifically, Chemero says: “*I am normally not aware of anything about my climbing abilities or riser heights when I perceive that I can climb a step. Humans, however, can—with training, and when so inclined—perceive things about their abilities and the features of the environment*” (p. 191). Although both Chemero and us try to conceptualize affordances in a relational context, our model puts the emphasis on the anticipatory and normative elements that characterize this relation (perceptual content). Agent’s abilities and norms in general are just agent’s features, some of which seem invariant (e.g., anatomy) and some of which could be developed or lost (e.g., skills, beliefs, etc.) through time. The invariants of the environment are also features of the situation. But these environmental and agential relata are not sufficient to make a relation (perceptual content) anticipatory and normative. The relation becomes anticipatory and normative, when the agent manages the respective interoceptive and exteroceptive inputs (constituent relata) by *evaluating* them with respect to the *consequences* they have for its norms (the anticipatory dimension) and by characterizing these anticipated outcomes as possible or not, right or wrong (the normative dimension). We should also note that it is not necessary for the agent to be aware of its abilities in order to proceed in the perception. The situation will dredge them up at the time of perception, in the sense that during perception the agent becomes aware of its interoceptive relata even though it could misinterpret them. Thus, we argue that the perpetual content (what is perceived) is an emergent value-rich anticipatory relation, which cannot be reduced to interoceptive plus exteroceptive perceptual processes. It is this anticipatory and normative relation that makes affordances interactive. In our understanding, *affordances are value-rich anticipatory relations that indicate an interaction potentiality*. Therefore, we would say that while an agent should in principle not be aware of anything about its climbing abilities in order to interact with the stairs, it becomes aware of itself when it perceives (i.e., forms an anticipatory relation) that it can “possibly” climb a step (the perceptual content) notwithstanding the fact that this content has always the possibility of being in error. This is opposed to explanations (see, e.g., Sauchelli, 2013), which are based on anticipation that presupposes established knowledge and experiences. Of course, training (learning) could reduce the possibility of error by supporting the perceptual processes in the future. To distinguish the above interpretation of affordances from other conceptions established in different frameworks than the one presented here, the concept of “*interactive affordance*” has been suggested to denote all those value-rich indicated potentialities of interaction that emerge in agent’s perception (Xenakis and Arnellos, 2013). Then, perception constructs interactive affordances.

For instance, a served table in a restaurant is not only a basic affordance for seating and eating. The whole situation affords anticipation for gathering and socializing in case the agent’s norms support such social interactions. So, gathering is an anticipatory relation and could be the content of perception. For example, anticipating a gathering involves values for both the indications (internal or external) that support this social interaction. These values exceed the information that is available through ambient light by a served table. Values are changing dynamically according to the norms of the agent and may form either anticipation for spending pleasurable moments, or for arguing with friends, etc. What a served table affords is not only a matter of the abilities of the agent but also a matter of its social norms (e.g., the agent knows that its friends are edgy and disagreeable by the end of the day) or other *optimal* norms (e.g., an *optimal* gathering is the one that everything will go smooth and fine). So, all these evaluated indications may influence the perceptual content thus altering the way an agent perceives and acts each time it interacts with a served table. For instance, an agent may perceive an annoying gathering (interactive affordance) through the table but it may also prefer this possibility in case its norms for friendship are stronger than its anticipation for an annoying gathering. Thus, in our view the content of perception (potentialities for action or interactive affordances) is neither permanent nor objective. It rather alters as the agent establishes its anticipatory relation to its environment. Based on the framework presented so far, the perception of interactive affordances presupposes normative content, which is dynamic and subjective.

As we’ve already mentioned, normative evaluation allows an agent to prepare itself for action by identifying the sources of success and error in a particular interaction. Preparation and evaluation plays an integral role in the construction of anticipation by affecting the way an agent perceives the interactive affordances in order to select the action the agent believes will best contribute to its goals (Wenke and Fischer, 2013). So, anticipation is dynamically determined by certain evaluative outcomes with respect to a combination of internal and external conditions under which the interaction will succeed and will thus bring the agent closer to its goals.

The possibility of failure of the anticipation introduces *uncertainty in perception* with respect to the consequences of the interactive indications e.g., the agent does not know if the dinner table will surely result in another annoying gathering (even though it is possible). Agents perceive interactive affordances and make decisions that could be uncertain with respect to the optimal achievement of their initial goals. Interacting with an airplane’s cockpit is for everyone (but experienced pilots) an uncertain situation in the sense that non-trained persons are unable to anticipate the consequences of their actions in relation to choices that a pilot should make for a safe flight. Therefore, agents develop ways that will handle and reduce their uncertainty in perception. Learning (training) is a very important process that results in the reduction of such uncertainty in perception. Through learning, agents can develop ways to evaluate indications of potential interactions, to anticipate the result of their perceptual outcomes, to prepare themselves, and possibly to learn to modify their actions when things do not go well (Christensen and Hooker, 2000).

However, also agents perceive situations with which they are not familiar, and for which learning is not currently available. In these cases, agents could also develop other ways to reduce their uncertainty in perception. As we argue in the next section, “the aesthetic,” and particularly, “aesthetic” emotional values, emerge mostly in uncertain conditions where learning is not available and the agent’s competence in such interactive challenges does not presuppose an optimal interaction. It is proposed that the respective emotional processes are a crucial aspect of perception as they contribute to the reduction of uncertainty through the assignment of values to indications of potential interactions (interactive affordances), thereby facilitating meaningful patterns of activity.

### “AESTHETIC” EMOTIONS AND THEIR ROLE IN SENSE-MAKING

The majority of writers argue in favor of the involvement of basic emotional activity^12^ in any kind of aesthetic experience (Higgins, 2008; Prinz, 2011). Those who study the underlying activations in the human brain during aesthetic perception describe aesthetics on the basis of the gratification that someone feels during the interaction with objects (of art) involving mainly emotional pathways (Barry, 2006; Cela-Conde et al., 2011; Chatterjee, 2011). Additionally, the anticipation of the impact of choices on the basis of reward values is detected in brain areas that are mostly known for emotional processing during an aesthetic experience (Cinzia and Vittorio, 2009; Grabenhorst and Rolls, 2011). In most of these studies, including those from an evolutionary point of view, authors should reconsider the distinction between art and non-art, based on results demonstrating that the brain areas processing the aesthetic response to art overlap with those involved in any adaptive perception (Brown et al., 2011; Rolls, 2011). Because of this, it seems that emotions are involved in any activity related to “the aesthetic” (Brown et al., 2011; Xenakis et al., 2012). As we argued in section “Perception Within a Normative Account of Sense-Making,” a normative account of “the aesthetic” presupposes that the content of aesthetic perception is evoked in order to play a specific role in making sense of the current situation. So, an important step toward an explanation concerning aesthetic perception is to ground “the aesthetic” (and its role) during sense making in emotional functionality.

Almost all theorists of emotions claim that pleasure and pain have a strong influence on adaptive behavior (Nelissen et al., 2007; Brehm et al., 2009). Roughly speaking, an abstract description of emotions normally consists of a type of processing that analyses the stimulus, and then, through an evaluative process, it signals other processes that control actions and plans (see, e.g., Baumeister et al., 2007; Rolls, 2011). Emotional evaluations detect opportunities and threats, as well as the potentiality of an interaction. Hence they influence the agent with respect to how it should act in a given interaction. The normative function of emotions is the elicitation of signals that emerge from the prospects for norm satisfaction or failure (Rolls, 2011).

In general, we can say that emotions play a major role in sense making: an active contribution to the *preparation* for establishing a relation between the agent and its environment (Xenakis and Arnellos, in press). As already mentioned, meaningful patterns of activity require the construction of value-rich relations between the agent and its world. Emotions fulfill this constructive role. Several quite recent accounts in the field of neuroscience and psychology share the idea that emotions receive inputs not only from sensory modalities, but also from the internal milieu they continuously monitor, thereby providing the agent with real-time inputs regarding the whole state of its body (interoception; Damasio and Carvalho, 2013). Neuroscientific evidence emphasizes the fact that during global emotional activations, these inputs enable comparisons of past, present, and future feelings, stored norms and expectations based on acquired internal models of personal or social behaviors (Craig, 2009). The outcomes of such evaluations are emotional signals that produce anticipatory values, whose intensity is a crucial aspect that influences the potential motivation to satisfy those norms. Emotional values thus are future-oriented. They are associated with indications of potential interactions, which accordingly suggest the agent should remain neutral, or, alternatively, to move toward the incentives and away from threats. Specifically, positive emotional values are associated with indications that correspond to the satisfaction of a norm (the agent is doing fine or perhaps better with something than it needs to). They form perceptual contents that allow the agent to anticipate a reward from the execution of their interaction plans. In contrast, emotions with negative values are associated with indications that correspond to interaction failure, in the sense that they are elicited when the agent anticipates problems with the satisfaction of its norms (the agent is doing worse than it needs to; Pugh, 1979; Carver, 2001; Prinz, 2011; Xenakis et al., 2012).

It is also widely accepted that such emotional evaluations could influence the anticipatory system in cases in which there is no relevant history that the agent could recall, while it attempts to resolve its perceptual uncertainty (Rolls, 2011; Grupe and Nitschke, 2013). This is strongly supported by experimental evidence, which shows that emotional evaluations do not occur when everything is familiar in an ongoing interaction. On the contrary, in uncertain situations, where a purely rational analysis of the available choices is insufficient, decisions are made mainly on the basis of emotions (see e.g., Bechara, 2004; Dalgleish, 2004; Bar, 2009; Craig, 2009; Singer et al., 2009). Emotional values are elicited so as to prepare the agent to decide between potential interactions through the development of strategies for the reduction of its uncertainties and the control of its experience (Pessoa, 2008; Heilman et al., 2010). Activations in a circuit running through brain areas involved in emotional processing (the orbito-frontal cortex (OFC) – the anterior cingulate cortex (ACC) – amygdala – OFC) show that emotions provide the agent with an updated feedback system that uses emotional values to reform anticipation. Through this feedback system, emotions perform a cost-benefit analysis of new signals that are compared to existing anticipation (Hampton et al., 2007; Pessoa, 2008; Grabenhorst and Rolls, 2011), thereby providing a continuously updated preparatory system (Paton et al., 2006). Each time those circuits are used there is a possibility for better predictions and the perception of the object becomes progressively less uncertain, while, simultaneously, the agent develops progressively more functional meanings (Stapleton, 2013). In other words, (and in accordance to Damasio and Carvalho, 2013), the emotional state according to which the agent *feels like* is possible (or not) to engage in an uncertain interaction context, is formed indirectly as a content that describes a state of the body, and which is accessible to the organism in which it occurs, thereby providing subjectivity to the respective perceptual processes.

Both the provided framework on perception and the experimental evidence on emotional functionality described above are the conceptual and theoretical basis upon which we model and explain the minimal content of aesthetic perception and the respective potential preferences that are realized in everyday interactions.

### A MODEL FOR AESTHETIC PERCEPTION AND ITS MINIMAL CONTENT

According to the arguments made so far, in its minimal form “the aesthetic” is related to an emotional process that supports the agent to form meaningful patterns of activity, thus contributing to the resolution of uncertainty. The need of perceivers to reduce the interaction uncertainty when they have difficulties in detecting a potential interaction outcome is what gives rise to “aesthetic” emotional responses. Such responses are values evoked from evaluative processes of interoceptive and exteroceptive inputs in relation to self-defined norms and goals. According to the analysis in section “Perception Within a Normative Account of Sense-Making,” perception is understood as a preparatory process, during which the agent forms a value-rich anticipatory relation with its world by constructing interactive affordances. Then, in the proposed framework, *a particular perception should be considered “aesthetic” when emotional values are involved in the construction of the interactive affordances, i.e., when the detected indications of potential interactions are emotionally evaluated*. It is in this way that an agent comes to establish a feeling of preference with respect to its relation to the world. In other words, we suggest that agents perceive aesthetically when they feel like it is possible or not to engage in an uncertain situation. It follows that *the minimal content of aesthetic perception is an emotionally valued interactive affordance* on the basis of which the agent *feels like* is possible (or not) to interact in the current context. Let us explain all these through an example of an everyday interaction.

Consider the act of “posting a letter.” Let’s suppose that for a given agent, the proper or preferred way to post a letter (i.e., what characterizes the optimal realization of this action) is to hand the letter directly to a postal agent. In other words, the agent’s uncertainty for the respective act is at its minimum (or even null) when he will be able to arrive at the post office and hand the mail to a post officer. For whatever reason, when this goal cannot be satisfied optimally, the particular agent is uncertain for the respective action. It goes without saying that this optimal way of interaction is a personal and self-established norm of the agent. Quite incidentally, the agent did not make it on time and his alternative option is to use the mailbox^13^. In this example, we stipulate that the agent is uncertain about using the mailbox for “posting a letter,” and he will have to evaluate the indications of potential interactions with the mailbox. This is the moment when the agent tries to reduce its uncertainty by establishing a feeling of preference of those interactive indications that are mostly anticipated to bring it closer to its optimal way of posting. Since the agent’s knowledge about the consequences of the act of “posting a letter through a mailbox” is minimal or non-existent, the agent should then somehow manage to proceed in the interaction. Due to this uncertainty, the evaluation of these indications will have an emotional nature (see Perception Within a Normative Account of Sense-Making and “Aesthetic” Emotions and Their Role in Sense-Making for details), which will provide to the whole perceptual process an aesthetically dimension. As said above, the outcome of this emotional evaluation is a value that contributes (either partly or totally) to the anticipatory content by suggesting that the current situation (context-mailbox-agent) will afford or not the agent’s level of optimal posting. In this respect, the agent is about to establish a *feeling and thereby to construct the minimal content of* its *aesthetic perception of* the mailbox. For instance, a positive feeling would emerge when the interactive indications, which are numerous (e.g., the usage of the slot as the entrance for the envelope), would be emotionally evaluated as denoting the potentiality for the optimal realization of “posting a letter” (and this is the minimal content of this particular aesthetic perception). Now, since in our example the interactive indications of the mailbox are not compatible with the agent’s norms for optimal “posting,” it is highly likely that the interaction would end at this point in time, provided that the agent–through its perception–would have established a negative feeling that would not indicate an optimal posting. However, agents also have other evaluative mechanisms that contribute to the formation of their perceptual content (e.g., rational inferences based on habits, and/or analogy making, etc.). For instance, the agent could form a negative feeling for posting the letter using the mailbox but for whatever reasons it thinks it ought to do so. Therefore, in all but the cases where those other evaluative mechanisms are not available, the minimal content of aesthetic perception does not determine but rather influence the agent’s preference or/and final judgments/actions. This is because emotional and cognitive evaluations are integrated and equally contribute to the perceptual content in such complex behaviors. According to Pessoa (2008; 155), “emotion and cognition are only minimally decomposable.” “A decomposable system is one in which each subsystem operates according to its own intrinsic principles, independently of the others.” The minimal content of aesthetic perception is related to this particular aspect of the emotional subsystem, whose operational principles are independent from cognition during perception.

But let us extend the example. Imagine that this time the agent should definitely post the letter and finds two mailboxes when arriving at the closed post office. They are completely functionally identical with respect to their structural characteristics (e.g., same size, same slots, etc.), but one has a fresh coat of paint, which makes it look brand new, while the other is quite rusty but has a sticker depicting a person with a hat and a moustache facing toward the user and reaching out his hand. According to the classical conception of aesthetics, the painted mailbox would be possibly perceived as “pretty.” However, according to the norms of our agent, the fact that something looks brand new and shiny does not imply an optimal interaction with it (e.g., think for instance someone that for whatever reason refuses to buy well-polished shined apples from the grocery shop or a well-painted second-hand car). On the contrary, there is a high possibility that our agent will choose the second mailbox as, notwithstanding his uncertainty, he evaluates the respective indications (e.g., the depicted postman) as affording a much more optimal interaction, always according to his norms. More specifically, due to uncertainty, the evaluation of the interactive indications are also of an emotional nature, and the respective outcome could be a positive feeling because the agent would see in the sticker the postman he would like to have been able to interact with in the first place.

In either case, when the agent decides (for whatever reason) to use the mailbox and the letter finally reaches its destination, the respective choice will be characterized as successful, thereby providing the act of “posting a letter through the mailbox” a new meaning. This feedback loop provides the agent with the capacity to learn from its actions, to accordingly trust (or not) its feelings, and consequently to form more elaborated evaluation in the future. Recurrent successful interactions with similar mailboxes may reduce the uncertainty of interaction with them and most likely will change the optimal way of “posting a letter” by constructing the respective preference.

It is clear in our example that we argue against “existing” or “established” aesthetic phenomena, but on the contrary, we consider these phenomena as emergent. Our claim is that an aesthetic perceptual content is a matter of the agent, it is dynamic and it is altered as the situation changes. Therefore, we oppose arguments that ground aesthetic perception in an externally imposed content. In this respect, there are no “aesthetic activities” *per se*, but there is a type of perception that can be considered to fulfill minimal requirements for what could be inter-subjectively and (epistemologically) conceived as “aesthetic.” In this way, it would be wrong to talk about objects that are aesthetic *per se*. “*aesthetic*” *is considered the indications of potential interactions, when they are emotionally evaluated with respect to the way they serve (the degree of fulfillment with regard to the optimal) an interaction to the world*. Thus in a naturalized context “the aesthetic” is nothing more than a way of interaction. It is a way to cope with the environment, and aesthetic perception functions (in a bio-cognitive manner) in the service of that coping. Accordingly, aesthetic interactive indications are not features or properties of the artifact but instead emerge as the agent decides to emotionally make sense of the current interactive situation. Thus, aesthetic perception provides the agent a normative way to determine if the interactive indications hold or not. Thus aesthetic perception is normative with a dynamic content. Specifically, when the mailbox is detected and evaluated positively or negatively (i.e., indicates the optimal, for the agent, “posting of a letter” or not), the anticipation for the outcome of the particular interaction is influenced, and the interaction uncertainty is reduced. This practically means that, whatever the reasons based on which the agent’s interaction with the mailbox could have been halted so far, the agent, through the aesthetic perception, is no more in a symmetrical relationship with the mailbox but it now constructs potential ways of interaction. Therefore, aesthetic perception influences the agent’s anticipation in a way that contributes to the reduction of interaction uncertainty, while it enhances the agent’s adaptability. No matter what, the indication of a “fresh coat of paint” has always the possibility of interaction failure, when, for instance, the slot is closed or the mailbox is full of letters. The “slot,” the “fresh coat of paint,” etc. are among an indefinite number of indications that influence the way an agent aesthetically perceives a mailbox as a means of “posting a letter.” This is what differentiates our perspective from others that relate the perception of “the aesthetic” to features of the object. Aesthetic perception is evoked not due to specific characteristics in the object, but because of the agent’s need to detect and evaluate indications of potential interactions with the object. Thus, interactive affordances and aesthetics are not two different aspects that work together during perception, but rather the aesthetic facilitates (and enhances) the agent’s ability to perceive interactive affordances (Xenakis and Arnellos, 2013).

As Gibson (1975; 320) has argued, it does not make sense to speak of two separate kinds of perception, an ordinary and an aesthetic one. Rather, “There is only one kind of perception, the perception of the world with the meanings and values already in it.” However, in our view, while there is one perception, the underlying processes available that contribute to the construction of the perceptual content vary. Several complex interrelated functional systems, when needed, serve the formation of a perceptual content in different conditions of interaction. According to the model we present here, aesthetic perception concerns those interactive cases (when uncertainty is high and there is no relevant knowledge available) in which emotions are elicited to evaluate the interactive indications.

In accordance with our model, experimental evidence indicates that, quite often, interactions with very low uncertainty render beholders bored after a while, and any aesthetic aspect of interaction gradually disappears (Carbon, 2011). On the contrary, when uncertainty is increased, aesthetic emotional activity is evoked again, which increases the possibility of new aesthetic perceptions. When such uncertainty is anticipated to be easily resolved, agents have more chances to form positive aesthetic perceptions. This could possibly explain the reason why objects, with which users are confronted, and which they characterize as innovative and novel, are also found to provoke mostly positive aesthetic perceptions (Tinio and Leder, 2009; Carbon, 2011). Additionally, experimental results demonstrate that when perceivers are exposed to objects prior to learning about their features, they can find them more attractive, even when the respective features result in decreased functionality. In contrast, when interaction uncertainty is increased to unacceptable rates (e.g., through conflicting visual and verbal information with respect to an object), negative aesthetic outcomes can emerge (Hoegg et al., 2010). As Carbon and Leder (2005) have argued, a combination of innovation and familiarity seems to have increased possibilities for positive aesthetic perceptions.

The consideration of “the aesthetic” as a way to cope and better understand the world by reducing the uncertainty of interaction allows us to rethink the division of perception between “pragmatic” and “aesthetic” forms. An understanding of “the aesthetic,” which is not associated only with art, but also underlies a practical engagement with objects, may not be obvious (Dissanayake, 1982; Schulkin, 2009) for it rejects, to a large degree, a well-established tradition. However, there are an increasing number of neuropsychological studies that support our argument concerning the relation between aesthetics and potentialities for action. Recently, Righi et al. (2014) provided evidence in favor of the hypothesis that aesthetic values allow perceivers to enhance the detection of potential actions and, consequently, the detection of motor affordances (see also Xenakis and Arnellos, 2013), showing that objects that are perceived to work better and to be highly attractive have a privileged and selective neural activation. Additionally, activations in brain areas related to preparation for action suggest a coupling between the perception of an object and the type of action that could possibly manipulate it (Baber, 2006). Moreover, these activations overlap with areas responsible for the emotional evaluations of the possible consequences of the potential action (Alexander and Brown, 2011; Etkin et al., 2011; Waszak et al., 2012).

Summarizing, what agents may perceive as aesthetic in an object is any combination of indications that could aid them in reducing their uncertainty of interaction and to accordingly feel like they are clearing their path toward a successful choice for an optimal interaction. This conception of “the aesthetic” provides a normative character to aesthetic perception setting aside any claim that considers its scope useless in everyday interactions.

## CONCLUSION

Several approaches and studies suggest a transcendental account of “the aesthetic.” These are concerned mostly with special features that objects should exhibit in order to provoke an aesthetic perception. This long tradition conceives “the aesthetic” in an externalist and art-centered context of rightness but fails to explain many phenomena that are intersubjectively taken to pertain to “the aesthetic.”

We have argued that all these phenomena should be dealt within a different theoretical and explanatory framework. We have provided a model of aesthetic perception, and in particular, we have suggested the organizational requirements for the emergence of its minimal content. The theoretical and conceptual basis allowed us to address the naturalistic grounding of important theoretical issues such as: (i) why agents form aesthetic responses during their interactions with objects, (ii) what are the conditions under which the aesthetic phenomenon occurs, and (iii) what constitutes a minimal content of aesthetic perception. Our model addresses also other issues such as (iv) whether it is legitimate to differentiate aesthetic perception from other forms of perception, and (v) the possibility of reducing aesthetic perception to the features of the object. Our intention was neither to explain aesthetic phenomena in their entirety nor to explain what an agent finally likes or dislikes based on judgments of preference or beauty. Thus we don’t deal with the outcome of particular aesthetic judgments, but we aim at providing an explanatory basis of how “the aesthetic” emerges and evolves in an agent.

Our model emphasizes the importance of a normative aesthetic perceptual content pertaining to its anticipatory nature, since the content specifies what is supposed to be perceived aesthetically (and here lies the normativity). The proposed model stresses that the aesthetic perceptual content is a matter of the agent, it is dynamic and it changes; therefore, it denies the existence of an externally imposed content or values. We don’t deny the importance of external invariants in the environment, but we consider them to play a supportive role, as the role played by the agent’s abilities and norms in the formation of an aesthetic perceptual content by the agent itself.

The strategy adopted here allows to overcome many theoretical and methodological problems that arise when naturalists attempt to explain complex aesthetic interactions (mostly with works of art) in the context of basic adaptive behavior and agency (see Brown and Dissanayake, 2009; Dissanayake, 2009; Brown et al., 2011; Bundgaard, 2014). In this way, the proposed model supports a general theory of aesthetic responses beyond works of art. Aesthetic perception is understood in the realm of social and communicative interactions, which do not necessarily result in positive or negative aesthetic judgments but promote comprehension through individual/personal and social cognition.

We proposed that a particular perception is considered aesthetic when an emotional evaluation with anticipatory features assigns values to interactive indications (interactive affordances) in situations that are mainly characterized by high degrees of uncertainty and minimal knowledge. In other words, aesthetic perception is the interactive state in which the agent emotionally *feels that it is possible (or not)* to interact in the current conditions. However, these emotional feelings do not necessarily determine the agent’s preferences and/or actions. Agents, according to their degree of autonomy and the respective bio-cognitive organization, are able to manage values and norms in favor of their most important goals and to act accordingly. In this respect, there are no “aesthetic activities” or “aesthetic objects” *per se*, but there is a type of perception that can be considered to fulfill minimal requirements for what would be inter-subjectively and (epistemologically not ontologically) conceived as “aesthetic.” Hence, what is aesthetically right or wrong should be associated to norms and emotionally evaluated interactive affordances that reduce uncertainty by clearing the path for successful interactions.

This model differs from any other explanation and model that deals with “the aesthetic” as an extra component of perception. However, a question that emerges from the above considerations is what type of agents has the capacity for these aesthetic perceptions? According to recent theoretical works, mainly in the realm of evolutionary biology, this question may find its answer in non-human agency (see, e.g., Prum, 2012; Killin, 2013; McCormack, 2013).

## Conflict of Interest Statement

The authors declare that the research was conducted in the absence of any commercial or financial relationships that could be construed as a potential conflict of interest.

## Footnotes

1 The broad term “object” is preferred to the term “artifact” for the description of the source of environmental stimuli. Such stimuli could refer to several categories of objects or events from different domains, such as material and immaterial objects (e.g., sounds, social events), living systems (e.g., other humans, animals), objects which are intentionally designed (e.g., tools, artworks) or natural objects (e.g., landscapes), etc.

2 The term “feature” is used to denote elements that constitute the structure of an object (see footnote 1) as the ones of visual/musical/literary patterns, substances, physical, or other kind of properties, geometrical relationships, functional characteristics, etc.

3 In our understanding, unattained potential interactions do not concern only all those preferred interactions, but as we will explain next (see footnote 11 in section “Perception Within a Normative Account of Sense-Making”) they also refer to ideal interactive outcomes that could be hardly accomplished. We call them *optimal ways of interaction.*

4 Interaction implies meaning and value about the current situation. A situation acquires meaning or makes sense, when triggerings are related to agent’s ongoing necessity for establishing a self-defined normative status, relative to its bio-cognitive organization and its goals. Sense making concerns endogenous activities, through which the agent constructs its relation with the world. These endogenous activities are what bring life to the world (for an extended analysis see Weber and Varela, 2002; Thompson and Stapleton, 2008; Froese and Di Paolo, 2011). Sense-making is purposive and functional but it does not always suggest action (Burge, 2009). However, sense making is fundamental for action selection, a process by which the agent differentiates between relations/situations and chooses the best for its norms and goals (Bickhard, 2006). We call these differentiated relations “meaningful patterns of activity.”

5 At this point, an obvious objection from the traditional aesthetician could be that whatever we prefer is not always what we consider as *beautiful*. Nevertheless, our aim is not to discuss the concept of beauty, because, as it is probably the most prominent of aesthetic concepts, it also must have been evolved during the various socio-cognitive interactions (see also below).

6 An agent interacts within an environment according to certain norms. Norms provide the agent with characteristic information about the relation between the ongoing interaction and the conditions for the maintenance of its organization closure (Christensen and Hooker, 2000; Bickhard, 2006). Normativity refers to the value attribution that is given to a process or interaction (e.g., adaptive or maladaptive to an interaction or process in an organism) with respect to a certain norm, i.e., with respect to whether a standard or level of possible performance—that is in some way adequate for a fulfillment of a function—is satisfied by that very process or interaction (see Barandiaran and Moreno, 2008; Burge, 2009).

7 As Cua and Fletcher (1975) have remarked long ago, all these theoretical or practical guidelines suggest a set of requirements for aesthetic rightness without providing any hint of how a set of features could be successfully embodied in an object, or how could it be successfully perceived in different contexts. Even works such as Birkhoff’s (1933) that propose mathematical tools for accomplishing the rightness for aesthetic properties such as balance, equilibrium, symmetry etc., and which have influenced several similar approaches, argue that it is not realistic to expect from these models to provide either a description and/or to determine aesthetic perception and evaluation.

8 The design process may be abstractly conceived as a future-anticipating activity that creates visions of a desirable future among groups of agents (Arnellos et al., 2007). Therefore, design should primarily be considered as a process of cognitive construction. In this perspective, the notion of “design” encompasses all relevant activities pertaining to the creation of a communicative medium, as it most prominently happens in the domains of humanities and liberal arts, human professions and services, creative and applied arts, and technology and engineering (Friedman, 2003).

9 *Instrumentality* focuses almost exclusively on the achievement of mainly behavioral tasks during an interaction with an object. The study of task achievement is very important in user centered analysis and evaluation techniques (e.g., usability testing), making the instrumentality of an object a major goal in the field of interaction design. In this context, a *hedonic* response is related to self-advancement, self-presentation, and other self-referential goals, where the perceived objects are media by which perceivers can express their selves and communicate (Hassenzahl, 2008).

10 In general, indications are considered to be relations between environmental conditions and potentially functional processes of an agent. For instance, a high environmental temperature could be an indication for sweating. For a detailed account of the notion of “indication” in the framework of Interactivism, see Bickhard (1993; 301). We note that for such an indication to hold the process of sweating and the respective “infrastructure” should be there and available to the agent even in the absence of the respective environmental conditions. This is a conditional relation between a sensory input and a value-rich indication of a potential interaction based on this input (Bickhard, 2006).

11 An optimal interaction is determined by the agent’s norms and is thus dynamic and ill-defined. Optimal interactions are not only the “preferred” ways of interaction,; they could also refer to ideal interactive outcome that could be hardly accomplished. For instance, a student driver may anticipate that after a few lessons he will be able to perform like a F1 driver. Although an “ideal” interactive outcome, since the student has minimal or no possibilities to accomplish it, it could nevertheless be considered an optimal way of interaction for the student. Moreover, what the student driver anticipates about the optimal driving of a car is different from what his or her instructor anticipates. It follows that in many circumstances, preferred and ideal ways of interaction will coincide. These personal optimal ways of interaction could be a reference point by which the student and the instructor evaluate their interaction possibilities and their progress in the course of action.

12 Basic emotions (Ekman and Friesen, 1971) are evolved in experiences or/and serve biological functions related to survival needs of the cognitive agent (Averill, 1994). Authors like Ortony and Turner (1990) are critical of the entire notion of basic emotions. However, as Fox (2008, p. 89) explains scientists have agreed among criteria that can be used to distinguish basic emotions from moods and feelings. Following Panksepp’s view, we use basic emotions as basic tools providing agents “with sets of intrinsic values that can be elaborated extensively via individual and cultural learning” (Panksepp, 2007; 1819).

13 The mailbox example was chosen to avoid complex description. One could think of an indefinite number of cases, where a user is anxious and feels awkward when it interacts with various kinds of machines such as checking-in devices, ATMs, ticket machines, but also contemporary electronic and electrical appliances, cockpits, etc.
